# Severe Hyponatremia Presenting with Minimal Symptoms

**DOI:** 10.7759/cureus.1830

**Published:** 2017-11-08

**Authors:** Anita Lwanga, Amira Mohamed, Mayank Patel, Andres Serrano

**Affiliations:** 1 Department of Academic Internal Medicine and Geriatrics, University of Illinois at Chicago; 2 Department of Critical Care, Icahn School of Medicine at Mount Sinai; 3 Department of Internal Medicine, Mount Sinai Hospital; 4 Department of Nephrology, Mount Sinai Hospital

**Keywords:** hyponatremia, thiazide, hypertension, hypertonic saline

## Abstract

Hyponatremia is a common electrolyte abnormality, however, encountering a patient with serum sodium level below 100 mEq/L and minimal symptoms is unusual. We present the case of an 86-year-old woman who was found to have serum sodium levels of 99 mEq/L. Her only complaint was difficulty in walking. On admission, and throughout her hospital stay, she did not have altered mental status, focal neurological deficits, or adverse outcomes. Her history, blood work, and urine studies pointed towards a diagnosis of thiazide-associated hyponatremia. Thiazide-associated hyponatremia can occur at any time during the course of thiazide administration. The first step that should be taken to manage this condition is discontinuing the medication. The lesson learned from this case is that the degree of hyponatremia does not always correlate with the severity of symptoms.

## Introduction

Hyponatremia is the most common electrolyte abnormality encountered in the inpatient and outpatient setting [1-3]. However, a patient presenting with severe hyponatremia, along with a serum sodium below 100 mEq/L, is uncommon.

## Case presentation

An 86-year-old woman presented with the chief complaint of difficulty walking. She lived with her daughter who agreed that she was moving more slowly than usual. At her baseline, the patient had memory lapses with regards to recent events but was able to complete most of her activities of daily living. The patient had a past medical history of hypertension, a right-sided thalamic infarct, and dyslipidemia. Her home medications included metoprolol, 50 mg daily, hydrochlorothiazide, 25 mg daily, lisinopril, 40 mg daily, aspirin, 81 mg daily, and simvastatin, 20 mg daily.

The patient was 4 foot 9 inches and weighed 88 pounds, which was 24 pounds below her previous weight documented five years prior. Her blood pressure was 233/122 mmHg, while the rest of her vital signs were within normal limits. She was alert, oriented to person and place but not time, and was able to provide most of her history. Her gait was slow, she had difficulty sitting up on her own, had decreased skin turgor, and did not have peripheral edema. The rest of her exam was unremarkable.

The initial workup done in the emergency department revealed a serum sodium of 99 mEq/L, a serum potassium of 3.7 mEq/L, serum chloride of 69 mEq/L, serum blood urea nitrogen (BUN) of 10 mg/dL, serum creatinine of 0.67 mg/dL, urine osmolality of 354 mOsm/kg, and urine sodium of 105 mEq/L. Her serum thyroid-stimulating hormone (TSH) and AM cortisol, which were measured later in the course of her hospital admission, were 0.66 uIU/mL and 21.5 mcg/dL, respectively. The patient was given 0.9% sodium chloride and was admitted to the intensive care unit. Five hours later, the patient’s neurological exam and laboratory studies were unchanged from admission (Table 1). At this point, the decision was made to switch from 0.9% sodium chloride to 3% sodium chloride. 

**Table 1:** The patient’s serum and urine laboratory values on admission, five hours later, and forty-eight hours after admission. BUN - Blood urea nitrogen. SI - International system of units.

	Admission	5 Hours Later	48 Hours Later
Serum Sodium	99 mEq/L	99 mEq/L	120 mEq/L
Serum Osmolality	214 mOsm/kg	216 mOsm/kg	-
Serum Potassium	3.7 mEq/L	3.5 mEq/L	3.8 mEq/L
Serum Chloride	69 mEq/L	70 mEq/L	95 mEq/L
Serum Bicarbonate	21 mEq/L	20 mEq/L	21 mEq/L
Serum BUN	10 mg/dL	8 mg/dL	8 mg/dL
Serum Creatinine (SI Units)	0.67 mg/dL (59. 2 mcmol/L)	0.53 mg/dL (46.9 mcmol/L)	0.61 mg/dL (53.9 mcmol/L)
Random Urine Sodium	105 mEq/L	118 mEq/L	-
Random Urine Potassium	34 mEq/L	26 mEq/L	-
Random Urine Creatinine	24mg/dL	16 mg/dL	-
Random Urine Osmolality	354 mOsm/kg	346 mOsm/kg	-

Within 24 hours of switching to 3% sodium chloride, the patient’s serum sodium increased to 109 mEq/L, and she did not have any changes in her neurological exam. The patient received hypertonic saline for a total of two days. At the end of the two-day period, her serum sodium increased to 120 mEq/L, her mental status remained intact, and her neurological exam remained unchanged.

## Discussion

We are presenting a case of an elderly woman who had minimal symptoms despite having a serum sodium of 99 mEq/L. Clinically, the patient appeared euvolemic. The differential diagnosis for euvolemic hyponatremia includes glucocorticoid deficiency, hypothyroidism, psychogenic polydipsia, beer potomania, low protein diet, and endurance exercise, all of which were not consistent with this patient’s history or serum studies; this left syndrome of inappropriate antidiuretic hormone secretion (SIADH) and an adverse reaction to a diuretic as possible causes [1, 3]. Thiazide-associated hyponatremia and SIADH share many features including a serum osmolality below 273 mOsm/kg, a urine osmolality above 100 mOsm/kg, and a urine sodium above 40 mEq/L [4]. The fact that patients with thiazide-associated hyponatremia are taking a diuretic makes it difficult to confirm the diagnosis of SIADH [4], as the diagnostic criteria of SIADH require lack of diuretic use for at least one week [1, 3]. The finding of inappropriately elevated urinary osmolality (354 mOsm/kg) and urinary sodium (105 mEq/L), with the medical history of hydrochlorothiazide intake, indicated that thiazide use was the most likely cause of her hyponatremia.

Thiazide-associated hyponatremia can occur within a few days or years of taking the medication [2-7]. Risk factors include older age, low body mass, and female gender [2, 4, 7]. Patients with thiazide-associated hyponatremia may have clinical features and a biochemical profile that is consistent with hypovolemic hyponatremia or euvolemic hyponatremia and can accordingly be divided into two subgroups [2, 4]. The first group appear to be volume-depleted and have an increased serum urea/creatinine ratio, a serum uric acid greater than 4 mg/dL, and a urine sodium greater than 20 mmol/L after discontinuing the thiazide [2]. The second group appear to be euvolemic and have a biochemical profile suggestive of SIADH with a low-normal serum creatinine, a low-normal serum urea, a serum uric acid below 4 mg/dL, and increased urine sodium [2]. In our patient’s case, her serum uric acid, which could have been helpful in determining which subgroup she fell into, was not checked. Furthermore, categorizing patients into one of the subgroups of thiazide-associated hyponatremia is difficult because patients often have biochemical features from both groups [2]. There are several case series of patients with thiazide-induced hyponatremia with serum sodium levels below 100 mEq/L; however, these patients were confused, obtunded, seizing, in a coma, or displayed some form of neurological abnormality [8-9]. The absence of severe neurological symptoms in our patient was very unusual. Though the exact mechanism by which thiazides cause hyponatremia has not been determined, it is likely that thiazides act by inhibiting urinary dilution at the distal renal tubules [2, 7].

The treatment of hyponatremia should be individualized and take into account the cause, acuity, and associated symptoms. Most authors agree that the first step that should be taken in managing thiazide-associated hyponatremia is discontinuing the medication [2-4], but there does not appear to be a consensus with regards to fluid administration. Verbalis, et al. recommends discontinuation of the diuretic, along with administration of hypertonic saline if the patient has an altered mental status or seizures, or discontinuation of the diuretic and administration of normal saline if the patient has mild neurological symptoms [3]. Liamis, et al. recommends discontinuation of the diuretic, along with an infusion of hypertonic saline in acute symptomatic cases, discontinuation of the diuretic, and water restriction in patients that have chronic euvolemic hyponatremia with minimal symptoms, or discontinuation of the diuretic and normal saline in patients that are hypovolemic [2]. On the other hand, Burst, et al. recommends continuation of the thiazide, along with an infusion of hypertonic saline for the first day to prevent over-rapid correction, or discontinuation of the thiazide and infusion with normal saline, along with fluid restriction, for less severe cases [7]. Based on Sterns’ recommendations, our patient’s thiazide was discontinued, and she was treated with hypertonic saline because of the severity of the hyponatremia [4] and failure to correct after a trial of normal saline. In order to avoid severe neurological complications, we attempted to increase the patient's serum sodium by 4 to 6 mEq/L every 24 hours [2]. During this period, her neurological status and electrolytes were closely monitored in the intensive care unit to prevent rapid correction of the hyponatremia, which can cause adverse neurological outcomes [4].

## Conclusions

We described the course of an atypical case of severe hyponatremia in the setting of thiazide use that was managed with 3% sodium chloride. Fortunately, the patient did not have focal neurological deficits or experience adverse outcomes during the course of treatment. The lesson that can be learned from this case is that the degree of hyponatremia does not always correlate with the severity of symptoms. The optimal management of patients with severe asymptomatic thiazide-associated hyponatremia is an area that requires additional research.
